# Morphological Characteristics of the Posterior Wall Associated with Complex Acetabular Fractures: A Radiological Study Using 3D Software and Fracture Mapping Technique

**DOI:** 10.1155/2022/9212895

**Published:** 2022-03-24

**Authors:** Siyu Tian, Shaobo Liang, Zhongzheng Wang, Pengyu Ye, Yingchao Yin, Junran Li, Ruipeng Zhang, Kuo Zhao, Zhiyong Hou, Yingze Zhang

**Affiliations:** ^1^Department of Orthopaedic Surgery, Third Hospital of Hebei Medical University, Shijiazhuang, Hebei Province, China 050050; ^2^Department of Pelvic and Acetabular Surgery, Honghui Hospital, Xi'an Jiaotong University, Xi'an, Shanxi, China 712000; ^3^Department of Geriatric Orthopedics, Second Hospital of Tangshan, Tangshan, Hebei Province, China 063000

## Abstract

**Background:**

The aim of the study was to compare the morphological distinctions of the posterior wall (PW) in different complex acetabular fractures using 3D software and fracture mapping technique and ultimately to provide for improved clinical treatment.

**Methods:**

One hundred and fourteen patients with complex acetabular fracture associated with PW were recruited. All patients were divided into two groups according to the injury mechanism of the PW: Group A (both-column and PW) and Group B (including posterior column and PW; T shape and PW; and transverse and PW). Fracture mapping was generated on the intra- and extrasurface of a standard template. The radiological parameters including spatial displacement, articular surface area, articular range, marginal impaction, and multifragments of the two groups were compared.

**Results:**

The spatial displacement, intra-/extra-articular surface area, and start and end point in Group A were 10.9 mm (IQR, 8.4-15.2), 8.2 ± 2.6 cm^2^, 17.9 ± 5.3 cm^2^, 0.8° (IQR, -6.0-16.2), and 107.5° (IQR, 97.2-116.9), respectively. The results in Group B were 30.4 mm (IQR, 16.8-48.7), 4.1 ± 2.0 cm^2^, 10.6 ± 4.4 cm^2^, 29.5° (IQR, 19.2-38.0), and 117.5° (IQR, 98.2-127.2), respectively. Marginal impaction was defined by Letournel et al. All the differences between two groups were significant (*P* < 0.05). The fracture map in Group A showed an “L”-shaped pattern and a “cusp” on the ilium, and the PW was located at 1/5 to 1/4 of the posterosuperior part of the acetabulum. The fracture maps in Group B were scattered and lacked consistency, and the PWs were confined to 1/10 to 1/8 of the posterior acetabulum.

**Conclusions:**

Quantitative measurements and fracture mapping represented the differences in morphological characteristics of PWs associated with complex acetabular fractures.

## 1. Introduction

Fractures of the posterior wall (PW) are the most common types of the acetabulum [1, 2]. It has been reported that the PW fractures may be isolated or associated with other acetabular injuries, including posterior column, T shape, transverse, and both-column fractures [3, 4]. Similar to acetabular isolated PW fractures, the first three PW fractures mentioned above are all caused by a direct strike posteriorly of the femoral head, which often involves marginal impaction, multifragments, and lesions of the femoral head [5]. In contrast, the injury mechanism of PW fracture in both-column fractures is created by a “pull-type” mechanism and often a large-sized fragment [3, 6]. Although the differences between the two types of PW fractures have been reported in recent literature [3, 7, 8], to our knowledge, there are no quantitative morphological comparisons of two kinds of PW associated with complex acetabular fractures.

As computed tomography (CT) processing software has emerged, an imaging technique called “fracture mapping” described by Armitage et al. [9] was utilized to reveal the distribution of fractures and guide surgical planning. In the past decade, the combination of 2D/3D CT and fracture mapping has also been proven to be effective in understanding the injury mechanism and fracture classification and morphology [10, 11]. In addition, this technique was widely used in the acetabulum. Yang et al. [12, 13] established a frequency map of acetabular both-column fractures and quadrilateral fractures to help surgeons gain insights into the surgery. Cho et al. [14] evaluated the characteristics of isolated PW fractures of the acetabulum with a cohort of 51 patients to show fracture patterns and recurrent fracture zones.

The aim of the study was to compare the morphological distinctions of PW in different complex acetabular fractures using 3D software and fracture mapping technique and ultimately to provide for improved clinical treatment.

## 2. Materials and Methods

### 2.1. Subjects

**Figure 1:**
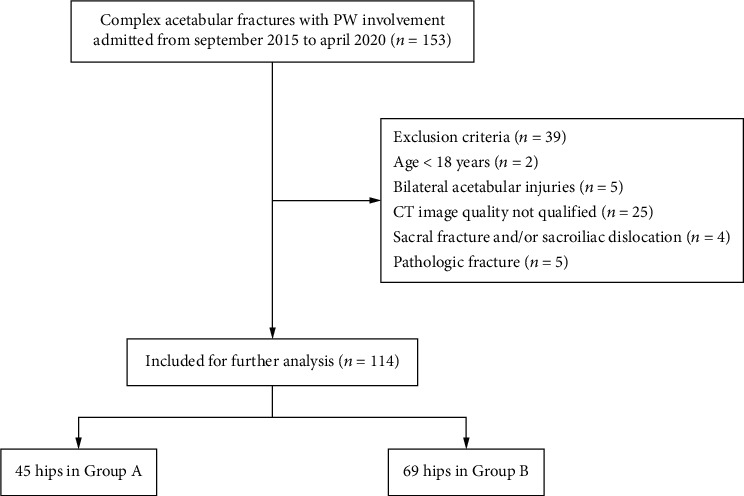
Flow diagram of included and excluded patients.

A retrospective study of patients with acetabular fractures was performed at our level I trauma center between September 2015 and April 2020. The inclusion criteria were as follows: (1) age of 18 years or older, (2) complex acetabular fractures with PW involvement, and (3) pelvic CT images with high quality and those with slice thicknesses of <1.5 mm. Exclusion criteria included (1) bilateral acetabular injuries, (2) sacral fracture and/or sacroiliac dislocation, and (3) pathological fractures. A total of 114 patients were identified in the picture archiving and communication system (PACS) database and classified by two trained orthopedic trauma physicians. The detailed inclusion and exclusion process is shown in Figure 1. All patients were divided into two groups according to the injury mechanism of PW: Group A (both-column and posterior wall, BC+PW) and Group B (including posterior column and posterior wall, PC+PW; T shape and posterior wall, T+PW; and transverse and posterior wall, TV+PW).

### 2.2. Radiographic Management

The CT scan Digital Imaging and Communication in Medicine (DICOM) files were imported into Mimics 20.0 software (Materialise Inc., Belgium). Referring to the method described by Yang et al. and Xie et al. [12, 15], a 3D model reconstruction was produced in which different fracture segments were distinguished in different colors. The 3D models were exported to 3-matic 12.0 software (Materialise Inc., Belgium). Using the sacrum and contralateral hip as a template, a mirrored hemipelvis was generated. All segments were manually reduced and automatically calibrated by “registration” to finish the reduction of the fracture.

### 2.3. Fracture Mapping

**Figure 2:**
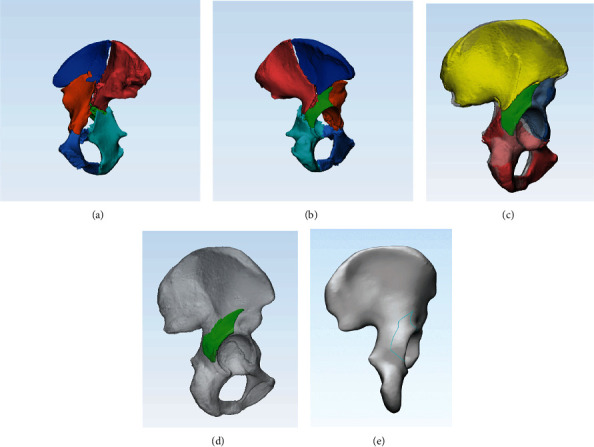
The procedure of fracture mapping: an example of BC+PW. (a, b) The 3D model was reconstructed, and different colors were used to distinguish the fragments. (c) The fragments were reduced based on the mirrored hemipelvis. (d) A standard template superimposed the reduced PW fragment. (e) The smooth curve was drawn on the standard template.

A 3D fracture mapping technique previously described by Xie et al. [11] was used to best match a standard acetabular template. Smooth curves were drawn precisely on the intra- and extrasurface of the template. All the fracture lines were superimposed on the same template to show the distribution of fracture lines in PW (Figure 2).

### 2.4. Parameter Measurement

To compare the different types of PWs quantitatively, we measured the following radiological parameters using Mimics 20.0 and 3-matic 12.0 software.

*Spatial displacement*: this measure was defined as the furthest 3D distance of a point on the acetabular rim of the PW before and after reduction.

*Articular surface area*: the intra- and extra-articular surface area of the PW was measured.

**Figure 3:**
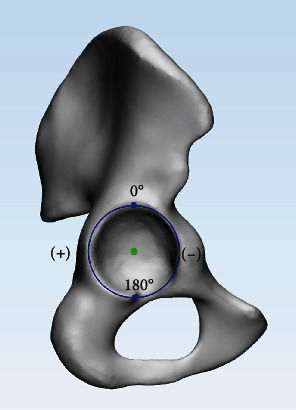
The clock was drawn in lateral view. The inferior point of the acetabulum was defined as +180°, and the superior point was 0°.

*Articular range*: as noted in the previous literature [14], a clock was drawn in the lateral view. The articular range of the PW was defined as the range between the start and end points on the rim and expressed in angle (°) (Figure 3).

In addition, marginal impaction and multifragments were recorded in each group.

### 2.5. Data Analysis

The analysis of the PW fracture maps was descriptive. All the data were processed using SPSS 23.0 (SPSS Inc., Chicago, IL, USA). Independent *t*-tests and Mann–Whitney *U* tests were used for the comparison of continuous variables. Differences in categorical variables were determined using the chi-square test. A value of *P* < 0.05 was considered statistically significant.

## 3. Results

The patient demographic characteristics and classifications are shown in Table 1.

### 3.1. Radiological Parameter Measurements

All parameters are presented as the median (IQR), the mean and standard deviation, or the proportion in Table 2 (box and whisker plots in Supplementary File). The median spatial displacements were 10.9 mm (IQR, 8.4-15.2) in Group A and 30.4 mm (IQR, 16.8-48.7) in Group B. The mean intra-articular surface area in Groups A and B was 8.2 ± 2.6 cm^2^ and 4.1 ± 2.0 cm^2^, respectively. The average extra-articular surface area in the two groups were 17.9 ± 5.3 cm^2^ for Group A and 10.6 ± 4.4 cm^2^ for Group B. The median start points of the articular range in Groups A and B were 0.8° (IQR, -6.0-16.2) and 29.5° (IQR, 19.2-38.0). The median end points in patients in Groups A and B were 107.5° (IQR, 97.2-116.9) and 117.5° (IQR, 98.2-127.2), respectively. The differences were statistically significant between patients in the two groups (*P* < 0.05).

Regarding marginal impaction, several patients in Group B had marginal impaction (18, 26%), whereas no patients in Group A had marginal impaction (*P* < 0.05). The differences in multifragments of patients in Group A (3, 7%) and Group B (36, 52%) were statistically significant (*P* < 0.05).

### 3.2. Fracture Map

**Figure 4:**
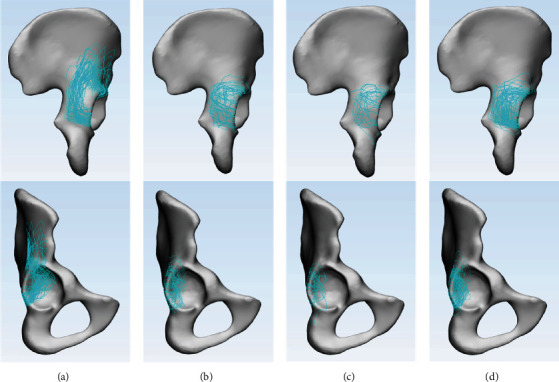
Extra- and intra-articular surface fracture maps of PWs: (a) BC+PW fracture; (b) PC+PW fracture; (c) T+PW fracture; (d) TV+PW fracture.

**Figure 5:**
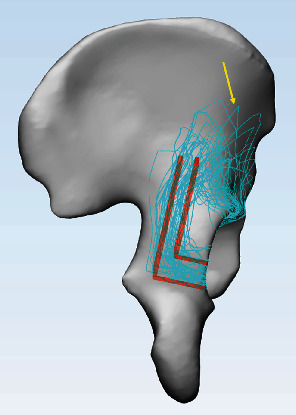
The extrasurface fracture map of BC+PW fracture. The high density of the two corridors formed a red “L”-shaped pattern. The “cusp” formed by the highest point of PW is marked with a yellow arrow.

In Group A, the inferior fracture lines of the PW ran approximately transversely from the acetabular rim to the medial wall. Then, the medial fracture lines progressed upward parallel to the acetabular rim (Figure 4(a)). The high density of the two corridors formed an “L”-shaped pattern (Figure 5). All the fracture lines extended upward beyond the level of the greater sciatic notch, continued upward toward the iliac crest in 34 patients (76%), and oriented around the anterior superior spine anteriorly in 11 patients (24%). The highest point of the PW formed a “cusp” on the outer table of the ilium (Figure 5). Moreover, the intra-articular fracture line revealed that the majority of PWs were located at the posterosuperior part and occupied approximately 1/5 to 1/4 of the acetabular fossa (Figure 4(a)).

The extra-articular fracture maps in Group B demonstrated that the majority of fracture lines were concentrated in the middle-lateral 3/4 of the PW; these fracture lines were scattered and lacked consistency (Figures 4(b)–4(d)). In addition, the main fracture lines were concentrated in 1/10 to 1/8 of the posterior acetabulum (Figures 4(b)–4(d)).

## 4. Discussion

Previous studies have widely reported radiological findings of the PW of the acetabulum. Some investigators described various methods for measuring the size and features of the PW to predict hip stability [3, 16–20]. However, these methods were confined to 2D and did not provide a 3D evaluation. A novel fracture mapping technique was applied by Cho et al. [14] to evaluate the features of isolated PW fragments, while PWs associated with complex acetabular fracture were not included. To our knowledge, this is the first work to compare the morphological characteristics of PWs associated with complex acetabular fractures using a combination of fracture mapping and 3D software.

**Figure 6:**
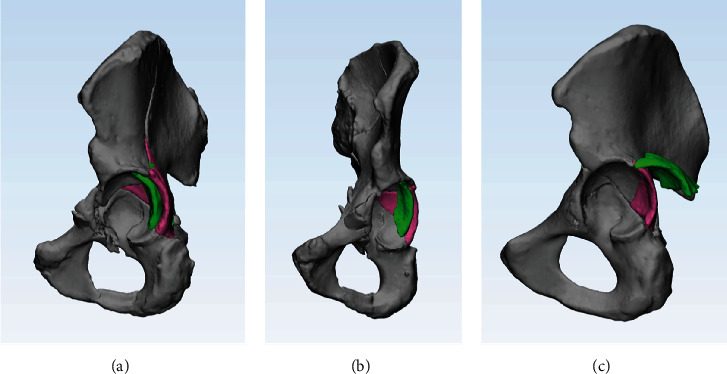
The direction of two types of PWs. Before and after reduction of PW is marked with green and pink, respectively. (a, b) The “pull-type” PW was displaced anteromedially in Group A. (c) The “posterior-type” PW was displaced posteriorly in Group B.

The differences in injury mechanisms resulted in the differences in the PWs observed between patients in the two groups. Both-column fractures frequently accompanied central dislocation of the femoral head. The force was transmitted medially to the main fragments, and the PW fracture was created by a “pull-type” mechanism anteromedially (Figures 6(a) and 6(b)), while the joint capsule was intact [3, 6, 21]. In contrast, the PW fractures in Group B were similar to acetabular isolated PW fractures, which were caused by a direct impact of the femoral head, posteriorly [4] (Figure 6(c)).

Except for the displacement direction of the PW, the displacement distances of Group A were significantly smaller than those of Group B (*P* < 0.05). Secondary congruency of the acetabulum and femoral head is a characteristic of acetabular both-column fractures in Group A, which means that the joint contact stress is evenly distributed over the entire articular surface [12]. And the displacement of PW in Group A disappeared following the reduction of the anterior column, quadrilateral plate, and femoral head [22]; this occurrence is known as a “congruency reduction.” However, after reduction of the main fragments (posterior column, T shape, and transverse) and femoral head in Group B, the visible displacement of the PW remained and required additional posterior fixation. It should be mentioned that displacements in this study were 3D distances, which were different from the measurements of the fracture gaps noted in radiographs or CT scans in other studies [23]. Therefore, the results reported in this study are quite different from those of other studies [24].

**Figure 7:**
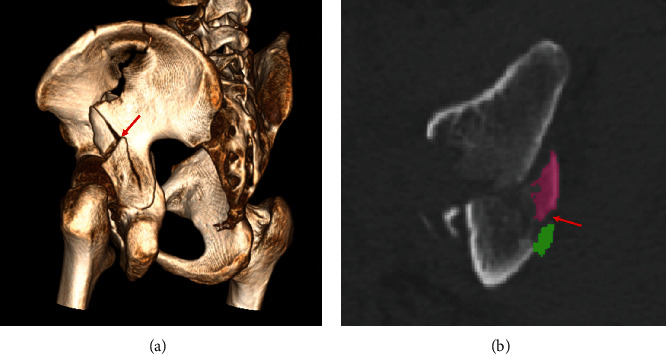
Two-part fragments of PWs in Group A. (a) 3D reconstruction shows that the fracture line (red arrow) did not involve the articular surface. (b) Two-part fragments are marked in green and pink. The fracture line (red arrow) did not involve the articular surface in axial CT sections.

The articular surface area and fracture map demonstrated that the PW in BC fractures were often a single large-size fragment. The main PWs in Group A occupied 1/5 to 1/4 of the acetabular fossa, in which the intra-articular surface area accounted for nearly 22% of the acetabulum. In addition, the results showed that the multifragments in Group A accounted for 3 of 45 cases (7%). All 3 cases were two-part intact fragments, and the fracture line did not affect the articular surface (Figure 7). In contrast, the smaller size in Group B took up approximately 1/10 to 1/8 of the acetabulum, which the intra-articular surface area was nearly 11% of the acetabular surface. Also, scattered fracture lines showed that 52% (36/69) patients had multifragments, even accompanied by intra-articular comminution of the PWs [4, 5], which was a related predictor for poor clinical outcomes [25].

**Figure 8:**
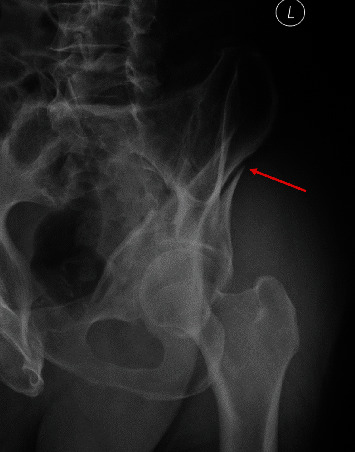
Radiographic characteristics of both-column fractures with PW involvement. The “antispur” sign is marked with a red arrow in the obturator-oblique view.

Furthermore, the location characteristics of the two types of PWs were also different. The articular range and fracture map reflected the PWs in Group A at a higher location, even involving the roof wall [26]. Eighty-seven percent (39/45) of the start points were between -25° and 25°, and 91% (41/45) of the end points were between 70° and 120°, whereas, in Group B, 91% (63/69) of the start points were between 0° and 50°, and 81% (56/69) of the end points were between 90° and 140°. This meant that the majority of the PWs in Group B were mainly confined below the roof [27], as described by Letournel and Judet. However, due to the uncertainty regarding the term “roof” [14] and obvious grouping differences between articles [14, 27], we did not divide the PWs of Group B into subgroups (posterior, posterior-superior, and posterior-inferior). Additionally, the fracture map of Group A elucidated a “cusp” of the PW on the outer table of the ilium. The “cusp” of the PW, which can be regarded as an auxiliary marker for diagnosing BC+PW fractures, was often obvious in the obturator-oblique view and was named the “antispur” sign [22] (Figure 8). In Group A, 76% fracture lines of PW extended upward toward the iliac crest and 24% oriented around the anterior superior spine anteriorly. This result demonstrated the morphological distinction of PWs in different AO/OTA subgroups (C1.3 and C2.3, respectively) and the incidence of the two subtypes, similar to the results revealed by Yin et al. [28].

Marginal impaction was defined by Cho et al. and e Souza et al. as a rotated, impacted fracture with depression of the osteochondral fragments into the underlying cancellous bone [14, 29], which was regarded as an independent risk factor for the development of arthritis and early clinical failure [5, 29, 30]. Our studies showed that 26% of “posterior-type” PWs had marginal impaction, which was similar to other results (21.5%-41.4%) [27, 31, 32]. However, because there was no direct impact on the surroundings of PWs in BC fractures, no marginal impactions were found in Group A.

All qualitative and quantitative morphological comparisons theoretically revealed the differences in posthip stability [33] and clinical prognosis [30] of the two types of PW. Combined with previous studies and our results, recommendations for the treatment of PW associated complex acetabular fractures were proposed. BC+PW fractures mainly involve the anterior stress of acetabulum and can be managed by a single ilioinguinal or iliac fossa combined with the Stoppa approach [8, 26, 34]. After reduction and fixation of the two columns, the posthip stability was evaluated using intraoperative dynamic stress examination [35]. Nonfixation or antegrade lag-screw fixation was determined based on evaluation results, and an additional Kocher-Langenbeck (KL) approach was unnecessary. The KL approach was obviously performed for the PWs in PC+PW, T+PW, and TV+PW. Furthermore, the intra-articular fragments and marginal impaction must be identified carefully and stabilized appropriately because these are the known factors affecting clinical outcomes [2].

This retrospective study had several limitations. First, there was a relatively small sample size gathered from one database. The paradox is that additional cases can improve the accuracy of the measured results; however, the increased fracture lines may lead to a complexity of mapping that is difficult to expound. Second, factors such as age, sex, and the force of impact endured upon injury were not taken into account. Third, because of the anatomical differences, some pelvis did not match the standard template perfectly.

In conclusion, 3D radiological measurements and fracture mapping represented the characteristics of PWs associated with complex acetabular fractures. There were significant differences among the three “posterior-type” PWs (posterior column, T shape, and transverse) and the PW associated with both-column fractures; also, the treatment for PWs should be different.

## Figures and Tables

**Table 1:** Patient demographic characteristics.

Variable	Group A^∗^	Group B^#^	*P* value
Mean age (years) (range)	47.6 ± 13.5	43.0 ± 12.3	0.062
Gender, *n* (%)			0.452
Male	39	64	
Female	6	5	
Side, *n* (%)			0.111
Right	29	34	
Left	16	35	
Mechanism of injury, n (%)			0.256
Vehicle accidents	19	40	
Fall from a height	21	23	
Others	5	6	

^∗^Group A: both-column and PW; ^#^Group B: posterior column and PW, T shape and PW, and transverse and PW.

**Table 2:** Comparison of parameters of PW in different fracture patterns.

Variable	Group A	Group B	*U*/*t*/*X*^2^	95% CI	*P* value
Spatial displacement (mm)^∗^	10.9 (8.4-15.2)	30.4 (16.8-48.7)	481.000	(-24.520, -13.570)	≤0.001
Articular surface area (cm^2^)^†^					
Intra-	8.2 ± 2.6	4.1 ± 2.0	9.216	(3.153, 4.879)	≤0.001
Extra-	17.9 ± 5.3	10.6 ± 4.4	8.008	(5.481, 9.084)	≤0.001
Articular range (°)^∗^					
Start point	0.8 (-6.0-16.2)	29.5 (19.2-38.0)	454.500	(-30.950, -18.870)	≤0.001
End point	107.5 (97.2-116.9)	117.5 (98.2-127.2)	1087.000	(-14.220, -2.830)	0.007
Marginal impaction, *n* (%)	0 (0)	18 (26)	13.940	(-0.365, -0.157)	≤0.001
Multifragments, *n* (%)	3 (7)	36 (52)	25.061	(-0.594, -0.316)	≤0.001

^∗^The values are given as median (IQR). ^†^The values are given as the mean and standard deviation. ^#^95% CI: 95% confidence interval of the difference.

## Data Availability

The data used to support the findings of this study are available from the corresponding author upon request.

## Supplementary Materials

Supplementary Materials Supplementary File: the box and whisker plots of the comparison of parameters of PW in two groups. A: spatial displacement (mm). B: intra-articular surface area (cm^2^). C: extra-articular surface area (cm^2^). D: start point of articular range. E: end point of articular range.
