# Supplementary material for: Morphological Characteristics of the Posterior Wall Associated with Complex Acetabular Fractures: A Radiological Study Using 3D Software and Fracture Mapping Technique
Source: Biomed Res Int. 2022 Mar 24;2022:9212895. doi: 10.1155/2022/9212895 (PMC8970878; doi:10.1155/2022/9212895)
Supplement: Supplementary Materials — Supplementary File: the box and whisker plots of the comparison of parameters of PW in two groups. A: spatial displacement (mm). B: intra-articular surface area (cm2). C: extra-articular surface area (cm2). D: start point of articular range. E: end point of articular range. [file 9212895.f1.docx]

**
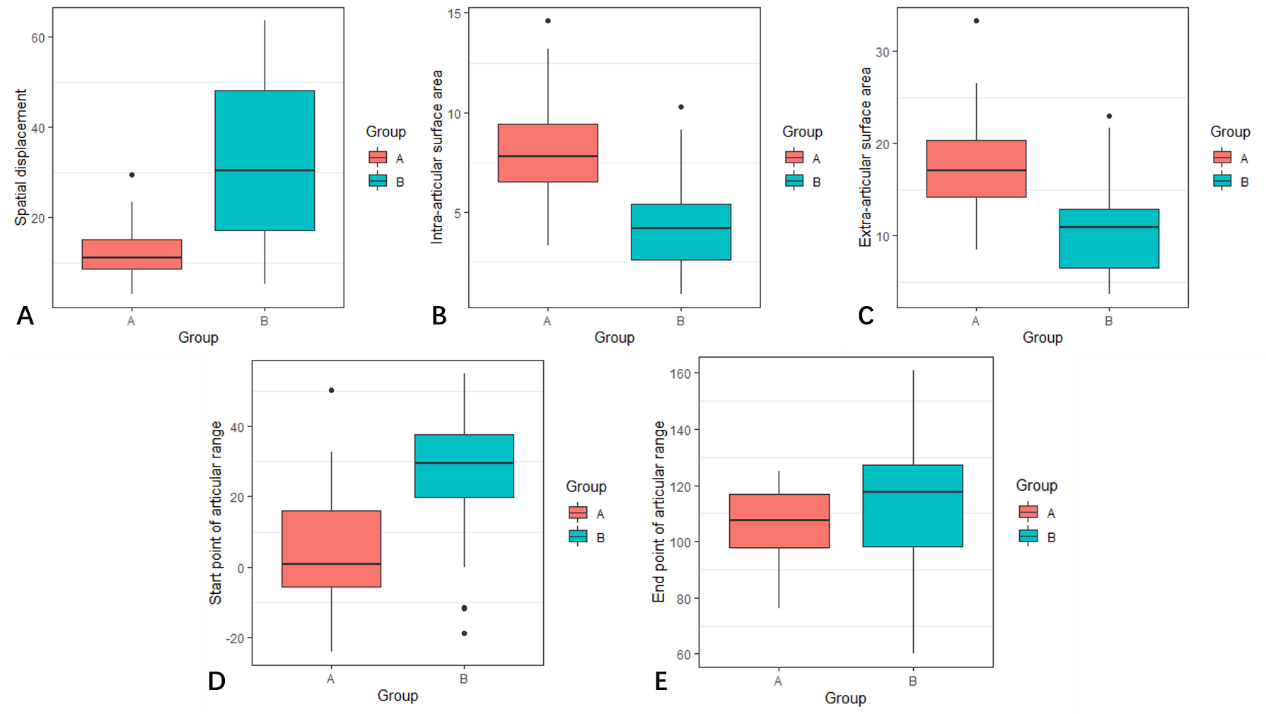
**

**Supplementary File.** The Box and whisker plots of the comparison of parameters of PW in two groups. **A.** Spatial displacement (mm) **B**. Intra-articular surface area (cm^2^) **C.** Extra-articular surface area (cm^2^) **D.** Start point of articular range **E.** End point of articular range.
